# Supplementary material for: Multi‐dataset identification of innovative feature genes and molecular mechanisms in keratoconus
Source: J Cell Mol Med. 2024 Sep 19;28(18):e70079. doi: 10.1111/jcmm.70079 (PMC11412914; doi:10.1111/jcmm.70079)
Supplement: Supplementary file 2 — Material S2. [file JCMM-28-e70079-s002.zip › 7.GSEA analysis/1.ARL11/KEGG_GLUTATHIONE_METABOLISM.html]

Details for gene set KEGG\_GLUTATHIONE\_METABOLISM[GSEA]

|  || Dataset | ARL11.group.cls#h\_versus\_l |
| Phenotype | group.cls#h\_versus\_l |
| Upregulated in class | l |
| GeneSet | KEGG\_GLUTATHIONE\_METABOLISM |
| Enrichment Score (ES) | -0.30211177 |
| Normalized Enrichment Score (NES) | -1.0037853 |
| Nominal p-value | 0.45039684 |
| FDR q-value | 1.0 |
| FWER p-Value | 1.0 |
Table: GSEA Results Summary

  

Fig 1: Enrichment plot: KEGG\_GLUTATHIONE\_METABOLISM      
 Profile of the Running ES Score & Positions of GeneSet Members on the Rank Ordered List

  

| SYMBOL | TITLE | RANK IN GENE LIST | RANK METRIC SCORE | RUNNING ES | CORE ENRICHMENT || 1 | GSTA3 | na | 2651 | 0.258 | -0.0080 | No |
| 2 | GSTA2 | na | 5278 | 0.158 | -0.0418 | No |
| 3 | GGT6 | na | 5919 | 0.139 | -0.0233 | No |
| 4 | GPX7 | na | 7609 | 0.100 | -0.0455 | No |
| 5 | GGT5 | na | 8131 | 0.090 | -0.0366 | No |
| 6 | GCLM | na | 8570 | 0.082 | -0.0276 | No |
| 7 | MGST2 | na | 10508 | 0.054 | -0.0691 | No |
| 8 | GGT7 | na | 11406 | 0.043 | -0.0837 | No |
| 9 | GSTT2 | na | 11478 | 0.042 | -0.0746 | No |
| 10 | MGST3 | na | 12526 | 0.031 | -0.0965 | No |
| 11 | GSTT1 | na | 13017 | 0.027 | -0.1035 | No |
| 12 | GPX1 | na | 14044 | 0.018 | -0.1282 | No |
| 13 | OPLAH | na | 15824 | 0.005 | -0.1782 | No |
| 14 | GGCT | na | 16286 | 0.002 | -0.1910 | No |
| 15 | GSTP1 | na | 17007 | -0.003 | -0.2109 | No |
| 16 | GSTO1 | na | 17080 | -0.004 | -0.2120 | No |
| 17 | GSTO2 | na | 17085 | -0.004 | -0.2112 | No |
| 18 | GSTK1 | na | 18167 | -0.012 | -0.2393 | No |
| 19 | GSTZ1 | na | 19033 | -0.017 | -0.2597 | No |
| 20 | GSS | na | 19104 | -0.018 | -0.2570 | No |
| 21 | RRM1 | na | 19697 | -0.022 | -0.2683 | No |
| 22 | GSTM5 | na | 20064 | -0.024 | -0.2724 | No |
| 23 | SRM | na | 20072 | -0.024 | -0.2662 | No |
| 24 | GCLC | na | 20481 | -0.027 | -0.2707 | No |
| 25 | SMS | na | 21570 | -0.036 | -0.2926 | Yes |
| 26 | TXNDC12 | na | 21732 | -0.037 | -0.2874 | Yes |
| 27 | GSTM3 | na | 21920 | -0.039 | -0.2825 | Yes |
| 28 | GPX4 | na | 22138 | -0.041 | -0.2778 | Yes |
| 29 | LAP3 | na | 22247 | -0.042 | -0.2696 | Yes |
| 30 | GPX3 | na | 22510 | -0.045 | -0.2652 | Yes |
| 31 | GGT1 | na | 22662 | -0.047 | -0.2571 | Yes |
| 32 | GPX2 | na | 22726 | -0.047 | -0.2463 | Yes |
| 33 | GSTM2 | na | 23101 | -0.051 | -0.2435 | Yes |
| 34 | GSR | na | 23356 | -0.054 | -0.2364 | Yes |
| 35 | PGD | na | 23664 | -0.058 | -0.2299 | Yes |
| 36 | GPX5 | na | 24047 | -0.063 | -0.2242 | Yes |
| 37 | IDH1 | na | 24481 | -0.069 | -0.2183 | Yes |
| 38 | G6PD | na | 24509 | -0.070 | -0.2006 | Yes |
| 39 | IDH2 | na | 25024 | -0.076 | -0.1951 | Yes |
| 40 | GSTA4 | na | 25863 | -0.088 | -0.1959 | Yes |
| 41 | ANPEP | na | 26145 | -0.093 | -0.1794 | Yes |
| 42 | RRM2B | na | 26702 | -0.102 | -0.1682 | Yes |
| 43 | ODC1 | na | 26879 | -0.106 | -0.1451 | Yes |
| 44 | RRM2 | na | 27737 | -0.124 | -0.1368 | Yes |
| 45 | GSTM1 | na | 28996 | -0.156 | -0.1316 | Yes |
| 46 | GPX6 | na | 30199 | -0.194 | -0.1146 | Yes |
| 47 | MGST1 | na | 31885 | -0.256 | -0.0953 | Yes |
| 48 | GSTM4 | na | 32561 | -0.292 | -0.0373 | Yes |
| 49 | GSTA5 | na | 33671 | -0.375 | 0.0305 | Yes |
Table: GSEA details [plain text format]

  

Fig 2: KEGG\_GLUTATHIONE\_METABOLISM      
 Blue-Pink O' Gram in the Space of the Analyzed GeneSet

  

Fig 3: KEGG\_GLUTATHIONE\_METABOLISM: Random ES distribution      
 Gene set null distribution of ES for **KEGG\_GLUTATHIONE\_METABOLISM**
